# Monte Carlo approximation of the logarithm of the determinant of large matrices with applications for linear mixed models in quantitative genetics

**DOI:** 10.1186/s12711-025-00991-1

**Published:** 2025-08-06

**Authors:** Matias Bermann, Alejandra Alvarez-Munera, Andres Legarra, Ignacio Aguilar, Ignacy Misztal, Daniela Lourenco

**Affiliations:** 1 Department of Animal and Dairy Science, University of Georgia, Athens, GA 30602 USA; 2 CDCB, Bowie, MD 20716 USA; 3 Instituto Nacional de Investigación Agropecuaria (INIA), 11500 Montevideo, Uruguay

## Abstract

**Background:**

Likelihood-based inferences such as variance components estimation and hypothesis testing need logarithms of the determinant (log-determinant) of high dimensional matrices. Calculating the log-determinant is memory and time-consuming, making it impossible to perform likelihood-based inferences for large datasets.

**Results:**

We presented a method for approximating the log-determinant of positive semi-definite matrices based on repeated matrix–vector products and complex calculus. We tested the approximation of the log-determinant in beef and dairy cattle, chicken, and pig datasets including single and multiple-trait models. Average absolute relative differences between the approximated and exact log-determinant were around 10^–3^. The approximation was between 2 and 500 times faster than the exact calculation for medium and large matrices. We compared the restricted likelihood with (approximated) and without (exact) the approximation of the log-determinant for different values of heritability for a single-trait model. We also compared estimated variance components using exact expectation–maximization (EM) and average information (AI) REML algorithms, against two derivative-free approaches using the restricted likelihood calculated with the log-determinant approximation. The approximated and exact restricted likelihood showed maxima at the same heritability value. Derivative-free estimation of variance components with the approximated log-determinant converged to the same values as EM and AI-REML. The proposed approach is feasible to apply to any data size.

**Conclusions:**

The method presented in this study allows to approximate the log-determinant of positive semi-definite matrices and, therefore, the likelihood for datasets of any size. This opens the possibility of performing likelihood-based inferences for large datasets in animal and plant breeding.

## Background

Likelihood-based inferences in quantitative analysis of traits in animal and plant breeding need logarithms of the determinant (log-determinant) of high dimensional matrices. Popular likelihood-based inferences in animal breeding and genetics are: parametric model comparison using the Likelihood Ratio Test or Akaike Information Criterion (AIC); using the value of the log-likelihood as a convergence criterion in expectation–maximization (EM) or average information (AI) REML; or optimizing numerically the log-likelihood function in derivative-free REML. The most computationally expensive step in likelihood-based inferences is the calculation of the log-determinant of high dimensional matrices. Thus, alleviating such a cost in terms of memory and time requirements would allow using large datasets and reduce the use of computing resources for likelihood-based inferences.

The matrices involved in likelihood-based inference are positive semi-definite. Thus, the (pseudo) log-determinant is calculated using the Cholesky (or LDL) decomposition of the matrix with a computing cost proportional to $O\left({n}^{2}\right)$, where $n$ is the dimension of the matrix. Although with sparse-matrix techniques the Cholesky decomposition is feasible for large matrices, it becomes time and memory-prohibitable for very large models or when genomic information is used, which considerably reduces the sparsity of the involved matrices [1]. Therefore, likelihood-based inferences cannot be made for large datasets or moderate-size datasets with genomic information. With frequentist or Markov Chain Monte Carlo (MCMC) methods, likelihood-based inferences are suitable in terms of time and memory usage for models with less than a couple of millions of equations. In contrast, large models for genetic evaluations could have tens or hundreds of millions of equations.

Since exact methods are unfeasible to apply to modern datasets with existing computing resources, numerical linear algebra is moving towards approximation methods for large applications. By employing notions of complex calculus, Hale et al. [2] and Aune et al. [3] approximated the log-determinant of a matrix $\mathbf{M}$ based on repeated matrix–vector products $\mathbf{M}\mathbf{x}$, where $\mathbf{x}$ is a suitable vector. In animal and plant breeding, there are very efficient algorithms for specific matrix–vector multiplications for extremely large matrices. In fact, most often these methods do not set up matrices explicitly. These algorithms involve iteration on data techniques [4–7] and specific methods for manipulating genomic matrices [8–10]. Therefore, approximating the log-determinant of these matrices by means of matrix–vector products seems feasible.

This study aims to introduce and adapt the approximation of log-determinants based on matrix–vector products and complex calculus ideas, which has already been used in other fields like physics, engineering, and spatial statistics, to the animal and plant breeding and genetics field. The approximation of the log-determinant will be compared against its exact counterpart, in terms of accuracy and computing time. Applications of the log-determinant approximation for variance components estimation will be tested and discussed.

## Methods

### Theory

In this section, we briefly review the theory for approximating $\text{log}\left|\mathbf{M}\right|$, where we assume that $\mathbf{M}$ is a positive semi-definite matrix. For more details, we refer the reader to Hale et al. [2] and Aune et al. [3].

**Fig. 1:**
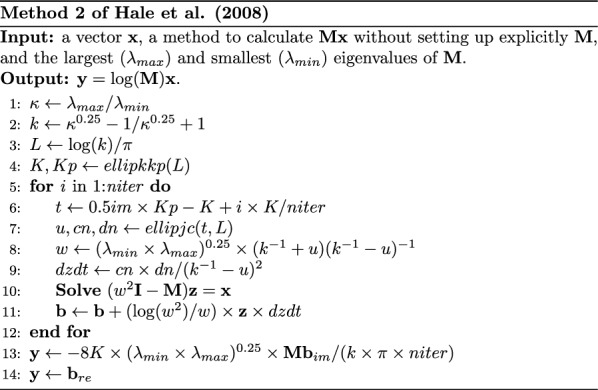
Algorithm for Method 2 of Hale et al. [2] for approximating the product of the logarithm of a matrix times a vector using complex contour integration. Matrices are in capital and bold font, vectors are in bold font, scalars are in italic font, and *im* refers to the imaginary unit or part of a complex number. In line 4, a complete integral of the first kind is evaluated. The three kinds of Jacobi elliptic functions are evaluated in line 8. The complex system of linear equations in line 11 is solved by the preconditioned conjugate orthogonal conjugate gradient method

Because of the Jacobi’s Determinant Lemma [11; p.309], we have the following identity:

1 $$\log \left| {\mathbf{M}} \right| = {\text{tr}}\left( {\log \left( {\mathbf{M}} \right)} \right)$$

where $\text{log}\left(\mathbf{M}\right)$ is the matrix logarithm (for a matrix with an eigendecomposition $\mathbf{M}=\mathbf{U}\mathbf{D}{\mathbf{U}}^{\mathbf{^{\prime}}}$, $\text{log}\left(\mathbf{M}\right)=\mathbf{U}\text{log}\left(\mathbf{D}\right){\mathbf{U}}^{\mathbf{^{\prime}}}$). Then, Eq. (1) is approximated by the Monte Carlo-based Hutchinson estimator [12] as:

2 $${\text{tr}}\left( {\log \left( {\mathbf{M}} \right)} \right) \approx \frac{1}{s}\mathop \sum \limits_{i = 1}^{s} {\mathbf{v}}_{i}^{\prime} \log \left( {\mathbf{M}} \right){\mathbf{v}}_{i}$$

where $s$ is the number of samples and ${\mathbf{v}}_{i}$ is a random vector taking values 1 or − 1 with a probability equal to 0.5. In this expression, the product $\text{log}\left(\mathbf{M}\right){\mathbf{v}}_{i}$ does not require explicit computation of the matrix $\text{log}\left(\mathbf{M}\right)$. Instead, the product is approximated using the Method 2 of Hale et al. [2] by solving the following integral based on Cauchy’s integral formula [13; p. 119]:

3 $$\log \left( {\mathbf{M}} \right){\mathbf{v}}_{i} = \frac{{\mathbf{M}}}{2\pi i}\mathop \int \limits_{{\Gamma }} z^{ - 1} \log \left( z \right)\left( {z{\mathbf{I}} - {\mathbf{M}}} \right)^{ - 1} {\mathbf{v}}_{i} dz$$

where $\Gamma$ is a closed contour in the region of analyticity of the natural logarithm function (all complex numbers but those on the negative real axis and zero) and looping once around the spectrum of $\mathbf{M}$ in the counterclockwise direction. Evaluating Eq. (3) involves estimating the largest $\left({\lambda }_{max}\right)$ and smallest $\left({\lambda }_{min}\right)$ eigenvalues of $\mathbf{M}$, evaluating a complete elliptic integral of the first kind [13, 14] and solving a system of linear equations with a complex coefficient matrix. Figure 1 shows the algorithm for calculating Eq. (3). Note that $\mathbf{M}$ is not required in Fig. 1, but a method to multiply $\mathbf{M}$ times a vector without explicitly setting up $\mathbf{M}$.

### Application details

We will focus the application on three kinds of matrices, which will be referred to indistinctly as $\mathbf{M}$:

- ${\mathbf{ZZ^{\prime}}}$ or ${\mathbf{Z^{\prime}Z}}$: GBLUP or SNP-BLUP matrices, where $\mathbf{Z}$ is the genotype content matrix [15, 16].
- $\mathbf{H}$: single-step GBLUP relationship matrices [17].
- $\mathbf{C}$: mixed-model equations matrices [18].

Let a linear mixed model be of the form $\mathbf{y}=\mathbf{X}\mathbf{b}+\mathbf{W}\mathbf{u}+\mathbf{e}$, where $\mathbf{y}$ represents the phenotypes, $\mathbf{X}\mathbf{b}$ the fixed effects, $\mathbf{W}\mathbf{u}$ comprises all random effects, and $\mathbf{e}$ is the error term. Assuming multivariate normality of $\mathbf{y}$ with $Var\left(\mathbf{u}\right)={\mathbf{G}}_{0}\otimes \mathbf{K}$, $Var\left(\mathbf{e}\right)=\mathbf{R}$, and $Var\left(\mathbf{y}\right)=\mathbf{V}=\mathbf{W}\left({\mathbf{G}}_{0}\otimes \mathbf{K}\right){\mathbf{W}}^{\prime}+\mathbf{R}$, the log-likelihood $\left(\text{log}L\right)$ and restricted log-likelihood $\left(\text{log}{L}_{r}\right)$ are:

4 $$- 2{\text{log}}L \propto \log \left| {\mathbf{R}} \right| + \log \left| {{\mathbf{G}}_{0} \otimes {\mathbf{K}}} \right| + \log \left| {\left( {{\mathbf{G}}_{0} \otimes {\mathbf{K}}} \right)^{ - 1} + {\mathbf{W}}^{\prime} {\mathbf{R}}^{ - 1} {\mathbf{W}}} \right| + {\mathbf{y}}^{\prime} {\mathbf{Py}}$$

5 $$- 2{\text{log}}L_{r} \propto \log \left| {\mathbf{R}} \right| + \log \left| {{\mathbf{G}}_{0} \otimes {\mathbf{K}}} \right| + \log \left| {\mathbf{C}} \right| + {\mathbf{y}}^{\prime} {\mathbf{Py}}$$

where a block of $\mathbf{K}$ could be equal to $\mathbf{Z}\mathbf{Z}\boldsymbol{^{\prime}}$ or $\mathbf{H}$, and $\mathbf{P}={\mathbf{V}}^{-1}-{\mathbf{V}}^{-1}\mathbf{X}{\left({\mathbf{X}}^{\mathbf{^{\prime}}}{\mathbf{V}}^{-1}\mathbf{X}\right)}^{-1}{\mathbf{X}}^{\mathbf{^{\prime}}}{\mathbf{V}}^{-1}$. The importance of approximating the log-determinant for the matrices above-mentioned follows from Eqs. (4) and (5).

Note that we do not cover the numerator relationship matrix (**A**) due to the simplicity of calculating its determinant (the product of Mendelian sampling variances). The key mathematical operation of the approximation method is the efficient multiplication of $\mathbf{M}$ times a suitable vector $\mathbf{x}$. The product ${\mathbf{ZZ^{\prime}x}}\;{\text{or}}\;{\mathbf{Z^{\prime}Zx}}$ can be obtained efficiently by storing $\mathbf{Z}$ as binary integers and performing a custom multiplication [10] or by more complex algorithms [19]. For single-step matrices, it is easier to calculate ${\mathbf{H}}^{-1}\mathbf{x}$ than $\mathbf{H}\mathbf{x}$ [20]; therefore, one should approximate $\text{log}\left|{\mathbf{H}}^{-1}\right|$, and then $\text{log}\left|\mathbf{H}\right|=-\text{log}\left|{\mathbf{H}}^{-1}\right|$. The efficient product ${\mathbf{H}}^{-1}\mathbf{x}$ could be carried out by different methods [8, 9, 21]. As pointed out by the associated editor, one could calculate $\text{log}\left|\mathbf{H}\right|=-\text{log}\left|{\mathbf{A}}^{11}\right|+\text{log}\left|\mathbf{G}\right|$, where ${\mathbf{A}}^{11}$ is the block of the inverse of $\mathbf{A}$ pertaining to the non-genotyped animals and $\mathbf{G}$ is the genomic relationship matrix. This decomposition yields from applying determinants of partitioned matrices to $$\mathbf{H}=\left(\begin{array}{cc}{\left({\mathbf{A}}^{11}\right)}^{-1}& -{\left({\mathbf{A}}^{11}\right)}^{-1}{\mathbf{A}}^{12}\\ 0& \mathbf{I}\end{array}\right)\left(\begin{array}{cc}{\mathbf{A}}^{11}& 0\\ 0& \mathbf{G}\end{array}\right)\left(\begin{array}{cc}{\left({\mathbf{A}}^{11}\right)}^{-1}& 0\\ -{\mathbf{A}}^{21}{\left({\mathbf{A}}^{11}\right)}^{-1}& \mathbf{I}\end{array}\right)$$. However, computing $\text{log}\left|\mathbf{G}\right|$ and $\text{log}\left|{\mathbf{A}}^{11}\right|$ in an exact way in large data sets is expensive. The latter, although sparse, needs to include all non-genotyped animals, which can be millions of individuals for many applications. Also, approximating separately $\text{log}\left|\mathbf{G}\right|$ and $\text{log}\left|{\mathbf{A}}^{11}\right|$ could be equally expensive as approximating $-\text{log}\left|{\mathbf{H}}^{-1}\right|$. Furthermore, for some iteration on data applications ${\mathbf{A}}^{11}$ might not be created (the product ${\mathbf{A}}^{-1}\mathbf{x}$ is done by reading the pedigree file). For those reasons we addressed directly the computation of $-\text{log}\left|{\mathbf{H}}^{-1}\right|$. Finally, the product $\mathbf{C}\mathbf{x}$ can be obtained with iteration on data [6, 7] plus the previous methods in case $\mathbf{C}$ includes genomic information.

For estimating the maximum and minimum eigenvalues of $\mathbf{M}$ (line 1 in Fig. 1), we use the Lanczos algorithm with full orthogonalization and implicit restarting [22; p. 562–570]. The Lanczos algorithm requires repeating $\mathbf{M}\mathbf{x}$ for some iterations to reduce $\mathbf{M}$ to a tridiagonal matrix $\mathbf{T}$. Then, the eigendecomposition of $\mathbf{T}$ is performed using the Intel MKL stev subroutine [23], and its maximum and minimum eigenvalues are an estimate of ${\lambda }_{max}$ and ${\lambda }_{min}$, respectively. The Lanczos algorithm is suitable for any symmetric matrix $\mathbf{M}$.

The evaluation of the complete integral of the first kind (line 4 in Fig. 1) and the Jacobi elliptic functions (line 8 in Fig. 1) was done by adapting and rewriting the algorithms of the ellipkkp and the ellipjc functions from Driscolls’ Schwarz-Christofel Toolbox [24].

The linear system $\left({w}^{2}\mathbf{I}-\mathbf{M}\right)\mathbf{z}={\mathbf{v}}_{i}$ needs to be solved in each iteration when approximating $\text{log}\left(\mathbf{M}\right){\mathbf{v}}_{i}$, where ${w}^{2}$ is a complex number (line 11 in Fig. 1). Since $\left({w}^{2}\mathbf{I}-\mathbf{M}\right)$ is non-Hermitian but symmetric, we chose the preconditioned Conjugate Orthogonal Conjugate Gradient method (COCG) [25], which involves one complex $\mathbf{M}\mathbf{x}$ product in each iteration.

The accuracy and speed of the approximation depend on the following parameters: number of Monte Carlo samples $\left({n}_{samples}\right)$, number of Lanczos iterations $\left({n}_{lanczos}\right)$, number of iterations for the integral approximation $\left({n}_{int}\right)$, and number of COCG iterations within each iteration for the integral approximation $\left({n}_{cocg}\right)$. As default, we set ${n}_{lanczos}, {n}_{samples, } {n}_{int, }\text{ and }{n}_{cocg}$ to 200, 5, 10, and 20, respectively. These values were empirically chosen based on a sensitivity analysis.

It is worth noticing that in animal and plant breeding many of the involved matrices are non-full rank. Examples are the incidence matrix of all linear models with more than one cross-classified effect; relationship (or covariance) matrices due to the presence of clones or identical individuals; non-blended genomic relationships when there are more individuals than loci. Therefore, the determinant of those matrices is zero and its log-determinant is not defined. For those cases, the log-likelihood requires the logarithm of the pseudo-determinant of $\mathbf{M}$ as $\text{log}\left|\mathbf{M}\right|=\text{log}\left|\mathbf{L}\mathbf{D}\mathbf{L}\mathbf{^{\prime}}\right|={\sum }_{i}{1}_{{d}_{ii}>0}\text{log}\left({d}_{ii}\right)$, where $\mathbf{D}$ is a diagonal matrix, and ${1}_{{d}_{ii}>0}$ is an indicator function that equals to one if ${d}_{ii}>0$ and zero otherwise [26; p. 527–528].

All algorithms were written in Fortran 90 and implemented in the Blupf90 software suite [27]. Computations were performed on a Dell PowerEdge R740XD server with 1.5 TB of memory, 45 TB of disk, and two Intel Xeon Gold 6258R processors, using four threads for the calculations. The sparse matrix computations, used as a benchmark, were carried out with the YAMS package [28].

### Materials

We tested the presented algorithm with two examples. In the first one, we show the efficiency of the method for approximating $\text{log}\left|\mathbf{C}\right|$ and $\text{log}\left|{\mathbf{H}}^{-1}\right|$ for various datasets. Also, the sensitivity of the method in terms of ${n}_{lanczos}$, ${n}_{samples}$, ${n}_{int}$, and ${n}_{cocg}$ was tested.

In the second example, we compared the exact and approximated restricted log-likelihood for a post-weaning gain (PWG) model with data provided by the American Angus Association (St. Joseph, MI). We also estimated variance components using two derivative-free methods with approximated log-likelihood and compared them against EM and AI-REML for the same dataset. The derivative-free method was used to check if the likelihood surface was correctly explored using the numerical approximation, i.e. if we arrived to the same optimum as using EM or AI-REML. It is worth noticing that EM and AI-REML do not use the approximation of the log-determinant presented in this work, because they optimize through first (and second in AI-) derivatives obtained by the Cholesky factor of $\mathbf{C}$, which also allows exact computation of the likelihood.

#### Example 1

We compared the log-determinant approximation versus its exact counterpart using four datasets, including dairy and beef cattle, chicken, and pigs. These datasets were already used and described in [1]. Table 1 shows the description of the datasets and the models used, including the number of equations, number of non-zero elements, and rank for $\mathbf{C}$ and ${\mathbf{H}}^{-1}$. Log-determinants were evaluated for previously estimated variance components. Also, the sensitivity of the method in terms of the number of samples and iterations for various steps was assessed.

**Table 1:** Description of the different datasets used to test the approximation of the log-determinant of the matrices

Dataset	Animal	Random effects	Traits	Number of animals^1^	$\mathbf{C}$			${\mathbf{H}}^{-1}$		
					Neq	Nnz	Rank	Neq	Nnz	Rank
1	Broiler	Animal + pe	4	213,297 (15,723)	876,904	1,988,086,395	860,498	213,297	124,211,802	213,297
2	Beef	Animal + mat + mpe	3	169,621 (10,000)	1,620,693	1,265,004,773	1,108,575	169,621	50,570,504	169,621
3	Pig	Animal + pe	4	987,080 (53,928)	4,406,864	23,303,667,982	4,329,957	987,080	1,456,467,650	987,080
4	Dairy	Animal + pe	1	9,408,140 (34,506)	19,302,524	716,066,375	19,170,955	9,408,140	627,421,998	9,408,140

Neq = number of equations, Nnz = non-zero elements in the upper triangle, $\mathbf{C}$=coefficient matrix of mixed model equations, $\mathbf{H}$=single-step relationship matrix, pe = permanent environmental effect, mat = maternal effect, mpe = maternal permanent environmental effect.^1^ Genotyped animals within parenthesis

#### Example 2

We used the log-determinant approximation method to calculate an approximated log-likelihood for a PWG model. Then, the approximated log-likelihood was compared to the exact log-likelihood for a range of heritabilities. The dataset had 169,621 animals in the pedigree, from which 76,758 had records, and 10,000 had available genotypes. The effects in the model were a contemporary group effect with 31,368 levels and the additive genetic effect. This dataset was also used for comparing a derivative-free REML with approximated log-likelihoods against EM and AI-REML. We chose Powell’s method [29] and Algorithm AS 319 [30] to minimize $-2\text{log}{L}_{r}$ (see Eq. (5)) and perform derivative-free REML.

## Results and discussion

Table 2 shows the accuracy and computing time for approximating the log-determinant of different matrices. The absolute relative difference between the approximated and exact log-determinant averaged 7.85 × 10^–3^, regardless whether the matrix was full rank or not. As expected, the approximation was slower than the exact method, which used sparse matrix techniques, when the dimension of the matrix was small or moderate (e.g., in the order of hundreds of thousands). However, for large-size problems with dimensions greater than a million, the approximation was 2 to 500 times faster than the exact method. The average empirical standard error of the approximations was 0.23% of the estimated value, except for $\mathbf{C}$ in dataset 3, where it represented 23.74% of the approximated log-determinant. This could possibly be caused by the high condition number of such $\mathbf{C}$ compared with the other matrices.

**Table 2:** Approximation^1^ of the log-determinant of $\text{C}$ and $\text{H}$ for the different datasets

Dataset	$\mathbf{C}$						${\mathbf{H}}^{-1}$					
	log-det			Running time (min)		$\kappa$^4^	log-det			Running time (min)		$\kappa$^4^
	Exact	Approximated^2^	Diff	Exact	Approximated^3^		Exact	Approximated^2^	Diff	Exact	Approximated^3^	
1	− 3,061,087	− 3,071,591 (7178)	3.43e−3	29	15 (2.4)	2.29e7	− 172,382	− 170,681 (913)	9.86e-3	0.5	7 (1.1)	2.15e6
2	− 5,050,909	− 5,018,819 (1604)	6.35e−3	651	8 (1.3)	1.67e6	− 114,985	− 115,124 (622)	1.21e-3	0.93	7.5 (1.3)	1.15e7
3	− 6,551,755	− 6,345,217 (1,506,665)	3.15e−2	1041	21 (2.5)	7.83e11	− 1,453,522	− 1,458,731 (1021)	3.58e-3	11.4	26.9 (4.1)	5.15e6
4^5^	− 6,930,682	− 6,937,059 (10,698)	9.20e−4	27,177	61 (10.45)	9.99e5	− 5,688,896	− 5,654,868 (3641)	5.98e-3	599	20.49 (3.3)	7.50e4

log-det = logarithm of the determinant, $\mathbf{C}$ = coefficient matrix of mixed model equations, $\mathbf{H}$ = single-step relationship matrix, diff = relative absolute difference between exact and approximated log-det. ^1^ Approximations based on 200 Lanczos iterations, 5 samples with 10 rounds to approximate the integral and a maximum of 20 iterations for COCG. Decimals were truncated. ^2^ standard deviations of the samples within parenthesis. ^3^ Includes Lanczos approximation and sampling. Average time per sample in seconds within parenthesis. ^4^ Matrix condition number. ^5^ The exact calculation of the log-det was carried out with 12 threads. The remaining calculations used 4 threads

**Fig. 2:**
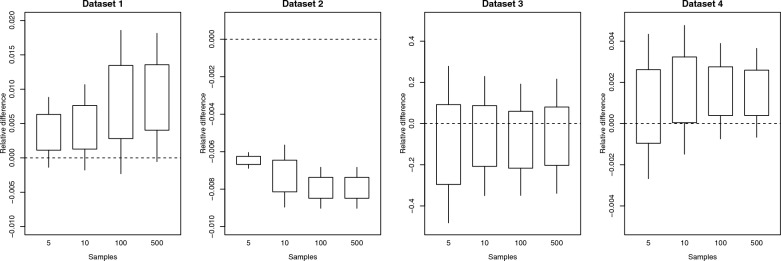
95% confidence interval for the relative difference between approximated and exact $\text{log}\left|\text{C}\right|$ for the different datasets when changing the number of Monte Carlo samples and keeping the rest of the parameters fixed to their default values

**Fig. 3:**
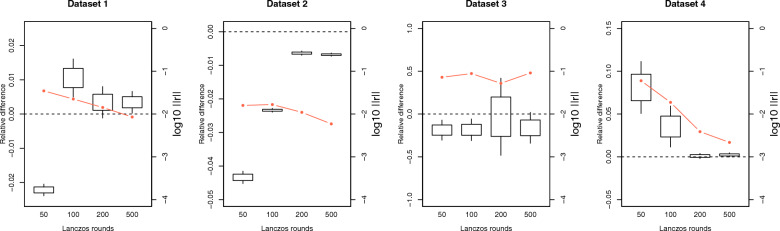
95% confidence interval for the relative difference between approximated and exact $\text{log}\left|\text{C}\right|$ for the different datasets when changing the number of Lanczos iterations and keeping the rest of the parameters fixed to their default values. The red line indicates the logarithm in base 10 of the norm of the residual for the smallest eigenvalue

**Fig. 4:**
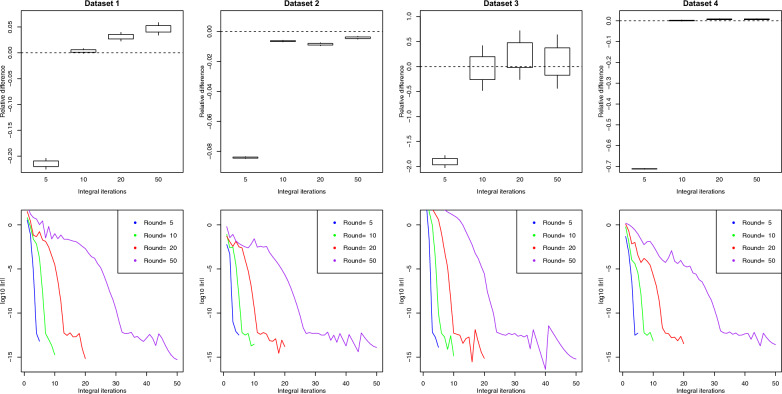
95% confidence interval for the relative difference between approximated and exact $\text{log}\left|\text{C}\right|$ for the different datasets when changing the number of iterations for the integral approximation and keeping the rest of the parameters fixed to their default values. The second line of plots shows the convergence of the COCG solver for the different iterations of integral approximation

**Fig. 5:**
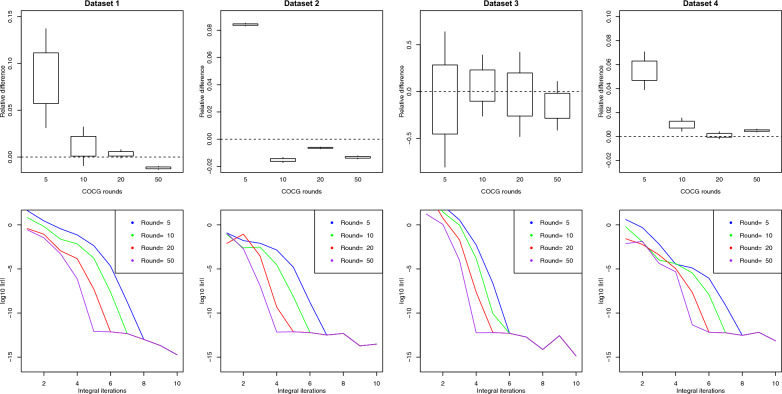
95% confidence interval for the relative difference between approximated and exact $\text{log}\left|\text{C}\right|$ for the different datasets when changing the number of COCG rounds and keeping the rest of the parameters fixed to their default values. The second line of plots shows the convergence of the COCG solver for each of the integral approximation iterations

As mentioned in “Application details” section, the accuracy and computing performance of the approximation depend on the following parameters: number of Monte Carlo samples $\left({n}_{samples}\right)$, number of Lanczos iterations $\left({n}_{lanczos}\right)$, number of iterations for the integral approximation $\left({n}_{int}\right)$, and number of COCG iterations within each iteration for the integral approximation $\left({n}_{cocg}\right)$. For computing purposes, it is desirable to keep those parameters to their minimum without compromising the accuracy of the approximation. The required time for approximating $\text{log}\left|\mathbf{M}\right|$ would be approximately $\left({n}_{lanczos}+{n}_{samples}{n}_{int}{n}_{cocg}\right)t$, where $t$ is the time taken by the multiplication of $\mathbf{M}$ times a vector. Figures 2, 3, 4, 5 show how the log-determinant approximation for $\mathbf{C}$ varies for each parameter while keeping the rest fixed to their default values.

For some datasets, the exact parameter is not covered by the empirical 95% confidence interval built from its estimator. The impact of this bias should be evaluated depending on the purpose of the log-determinant approximation. Previous uses of Hutchinson estimators in animal breeding were for the approximation of the trace of the product of two matrices for variance components estimation with REML [31–34], but not for computing likelihoods. However, in their studies, the authors did not compare the estimates of the traces against the true values. Therefore, any hypothesis regarding the bias in the approximation of the log-det should be assessed in future studies.

The number of Monte Carlo samples $({n}_{samples})$ employed in our study seems extremely small when comparing the proposed Monte Carlo estimator against MCMC sampling for variance components estimation. However, such small numbers were also successfully used to approximate the trace of the product of matrices for Monte Carlo REML [32–34]. The usage of small numbers of Monte Carlo samples for Hutchinson estimators arises from the low autocorrelation between successive samples which is a fact that does not occur when estimating variance components by MCMC methods.

**Fig. 6:**
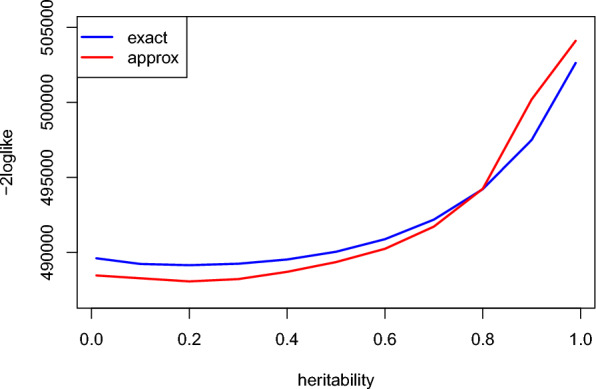
Comparison of exact and approximated log-likelihood of a post-weaning gain model (i.e., second example) for a grid of heritabilities

The results for Example 2 are presented in Fig. 6 and Table 3. Regardless of the difference between the exact and approximated likelihood in Fig. 6, it can be observed that both have a minimum at a heritability equal to 0.18, which is the value obtained when estimating variance components with EM or AI-REML. However, for estimating variance components with approximated log-determinant, only the DF-REML with Powell’s method [29] converged to the same heritability as EM and AI-REML. Although Algorithm AS-319 [30] was the fastest method, it overestimated the heritability by five points (0.23 vs. 0.18). This overestimation was also observed when the exact log-determinants were used in place of the approximated ones (results not shown).

**Table 3:** Comparison between expectation maximization (EM), average information (AI), and approximated derivative-free (aDF) REML for post-weaning gain in American Angus cattle

	EM-REML	AI-REML	aDF-REML (AS-319)^1^	aDF-REML (Powell)^1^
Rounds to convergence/function evaluations^2^	106	9	109	254
Time per round/function evaluation (min)	8.55	28.17	1.10	1.10
Total time (min)	907.51	254.75	130.9	282.4
Residual variance^3,4^	1726.9 (20.66)	1727.5 (20.66)	1665.9 (21.72)	1719.5 (21.51)
Additive genetic variance^3,4^	388.6 (24.89)	387.9 (24.89)	499.2 (28.19)	401.1 (26.41)

^1^ Optimization method. ^2^ For EM and AI-REML the first row denotes the number of rounds, whereas for the Approximated DF-REML denotes the number of likelihood evaluations. ^3^ Expressed in lb^2^. ^4^ Standard errors of the variances are within parenthesis

Typically, variance components estimation with large datasets is done by subsetting the original dataset. This practice could be problematic, for example, when the estimation of the correlation between two traits is sensitive to the history of selection on either one. In such a case, selecting a data subset that includes several generations of data is necessary to consider selection properly. This could generate a data subset that could still be very large for applying REML or MCMC methods. Furthermore, although approximated methods like the one presented in this study present numerical errors, they are usually based on mathematical and statistical properties and are easier to measure than errors arising from data subsetting.

The methods presented in this study allow likelihood-based inferences for large populations like the ones used in animal and plant breeding. Some, but not all, possible uses include parametric model validation like Akaike Information Criterion (AIC) or Bayesian Information Criterion (BIC), derivative-free algorithms for estimating variance components [35], calculating likelihood values to assess the convergence of Monte Carlo REML [34], and estimating relationships across metafounders without truncating the likelihood [36]. Although derivative-free methods are currently not used, the most popular used algorithms dated from the’60s [29, 37]; hence, new derivative-free algorithms with better convergence properties than older ones could be a future research topic. These new algorithms, coupled with log-determinant approximations could provide a computationally attractive way of estimating variance components where memory and running time make REML and MCMC methods unfeasible to apply. Current use of genomic selection and large size of historical datasets is leading to this situation. For instance, national dairy cattle evaluations have hundreds of millions of equations and more than one million genotyped animals [38, 39]. In plant breeding, the models have more traits but less equations and genotyped individuals than in animal breeding. In such setup, the computational cost of the methods used in plant breeding to address such peculiarity do not scale well when increasing the number of genotypes [40–42].

The exact calculation of log-determinant should still be preferred whenever possible. In cases where the exact calculation is not possible, it is necessary to note that errors due to the random sampling of the Hutchinson estimator occur during the approximation process. Even though the estimator is unbiased, the Monte Carlo error could cause bias in estimated parameters, especially when iterative algorithms are close to convergence. Previous studies have shown that reusing the same sequence of random numbers for each round of the Hutchinson estimator reduced the noise of the parameter’s estimates and the number of rounds until convergence [34]. Therefore, this might be a possible way of dealing with the statistical noise of the approximation of the log-determinant when using it with Monte Carlo REML or derivative-free algorithms. Furthermore, it would make sense to apply this technique when comparing models based on parametric statistics or for hypothesis testing so the random noise in different approximations is canceled out.

## Conclusions

We brought from other fields a method to approximate the log-determinant of a positive semi-definite matrix and adapted it to the field of animal breeding and genetics and, consequently, to plant breeding. The algorithm relies on successive matrix–vector multiplications, which can be done for linear mixed models in quantitative genetics due to custom efficient algorithms employed in routine genetic evaluations. The method can be applied to matrices of any size and could be used for Monte Carlo REML, derivate-free REML, parametric validation, and hypothesis testing. Approximating the log-determinant introduces random noise due to replacing an exact value with an approximated one. This error should be considered when using the proposed methodology.

## Data Availability

Data and material are available upon request.
